# Are Health Literacy and Patient Activation Related to Health Outcomes in Breast Cancer Patients?

**DOI:** 10.3928/24748307-20210524-02

**Published:** 2021-07

**Authors:** Chisom Kanu, Carolyn M. Brown, Karen Rascati, Leticia R. Moczygemba, Michael Mackert, Lalan Wilfong

## Abstract

**Background:**

Assessing health literacy and patient activation at the beginning of care could facilitate the provision of appropriate information to patients with breast cancer and increase the effectiveness of interventions geared toward improving patient involvement in self-managing their health and, consequently, their quality of life.

**Objective:**

The aim of this study was to evaluate cancer health literacy and patient activation in patients with breast cancer as well as examine their relationships to health-related quality of life (HRQoL) and resource use.

**Methods:**

Patients with breast cancer positive for human epidermal growth factor receptor 2 (HER2+) receiving care at 12 oncology clinics in Texas were offered participation in the study via convenience sampling. The survey consisted of the 6-item Cancer Health Literacy Tool, the 13-item Patient Activation Measure, the 27-item Functional Assessment of Cancer Therapy – General (version 4), and single-item measures for number of emergency department visits and hospitalizations as well as clinical and demographic characteristics.

**Key Results:**

The mean age of the 146 study participants was 57.1 ± 10.8 years; 92% (*n* = 134) had a high probability (≥0.7) of adequate cancer health literacy whereas 68% percent (*n* = 99) had high patient activation (level 3 or 4). Cancer health literacy had significant positive relationships with education and household income. Patient activation, education, and number of treatment types received explained 23% of the variation in HRQoL, and all except cancer health literacy were positive and significant predictors. No bivariate/multivariate analysis was conducted for emergency department visits and hospitalizations because there were few reported incidents.

**Conclusions:**

Interventions that aim to improve HRQoL in patients with breast cancer could target modifiable factors like patient activation. The homogeneity of cancer health literacy among study participants might have influenced its nonsignificant relationship with HRQoL and patient activation. Further assessments of health literacy and patient activation in larger and more diverse populations of patients with breast cancer are warranted. **[*HLRP: Health Literacy Research and Practice*. 2021;5(3):e171–e178.]**

**Plain Language Summary:**

In this study, the majority of patients with breast cancer were found to have high levels of cancer health literacy, patient activation, and health-related quality of life (HRQoL). The significant relationship between patient activation and HRQoL implies that patients with breast cancer who are able to actively participate in managing their health and health care are more likely to have higher HRQoL. Interventions that aim to improve HRQoL in patients with breast cancer could target modifiable factors like patient activation.

There is increasing recognition in oncology practice that a patient's quality of life, and not just quantity of life, is important to cancer treatment (Paraskevi, 2012). Despite the efficacy of current breast cancer treatment regimens, patients with breast cancer are usually burdened with physical symptoms and psychosocial distress from their condition, as well as unpleasant treatment side effects that adversely affect their health-related quality of life (HRQoL) (Perry et al., 2007; Susan G. Komen Foundation, 2016). Patients with cancer who are enabled to be more involved in monitoring their health have been shown to have significantly better HRQoL and fewer emergency department (ED) admissions (Basch et al., 2016). Furthermore, early detection of breast cancer recurrence in survivors significantly increases the chances of treatment success and survival (Lu et al., 2009).

A person's health literacy and ability to self-manage health (i.e., patient activation) are important factors that influence whether a patient knows if and when to seek care for symptoms instead of enduring a poor HRQoL, or has to visit the ED or be hospitalized for worsened symptoms. The Health Literacy Skills (HLS) framework put forth by Lee, Arozullah, and Cho (2004) describes how health literacy affects health outcomes through intermediate factors. Specifically, the HLS framework posits that people with lower health literacy are likely to have poorer medical knowledge, worse health behavior, less regular preventive care and physician visits, and poorer compliance with routine clinical visits and medications. These factors, in turn, may delay seeking timely and appropriate care, produce poor health outcomes, and increase the use of emergency and hospital services (Lee et al., 2004). Self-care is one of the mechanisms through which health literacy influences health outcomes (Paasche-Orlow & Wolf, 2007), and the factors that drive self-care (motivation, problem-solving, self-efficacy, knowledge, and skills) are embodied in patient activation (Hibbard et al., 2004). Thus, we cn expect that health literacy will affect health outcomes primarily through its impact on patient activation as posited by the HLS framework.

Health literacy pertains to a person's ability to obtain, communicate, process, and understand basic health information and services needed to make appropriate health decisions (Kindig et al, 2004), whereas patient activation refers to a patient's knowledge, confidence, and skills to facilitate active participation in self-managing health and health care (Hibbard et al., 2004). It is recommended that when examining the relationship between health literacy and health outcomes, factors that could confound (e.g., age, income, and health insurance status) and mediate (e.g., self-efficacy, self-care) the relationship should be closely examined (DeWalt et al., 2004). In fact, patient activation has been found to mediate the relationship between health literacy and resource use (Charlot et al., 2017). Although related, both constructs are distinct predictors of health and could be targets for behavioral intervention (Sheikh et al., 2016; Smith et al., 2013).

The few studies that have examined health literacy and patient activation were not exclusive to patients with breast cancer, used general health literacy measures, and/or did not directly examine the relationship between health literacy and patient activation. The aim of this study was to evaluate health literacy and patient activation in a sample of patients with breast cancer as well as examine the relationships of these constructs to health outcomes using an abridged version of the HLS framework (**Figure 1**).

**Figure 1.**
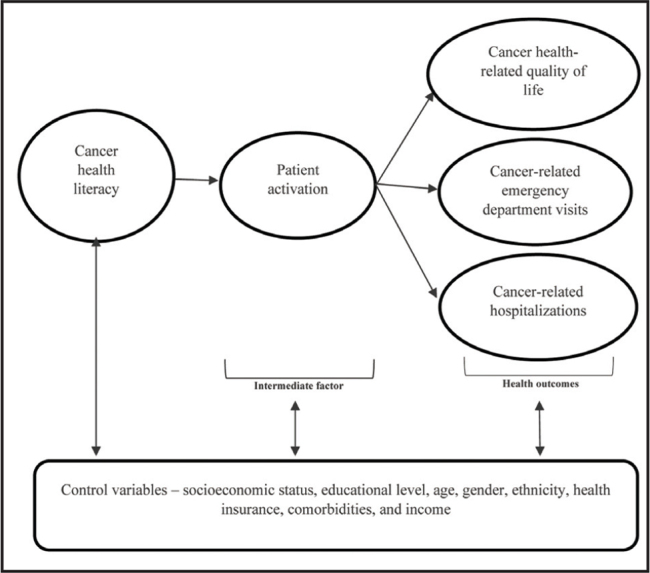
Health literacy skills framework in patients with breast cancer. Adapted from “Health literacy, social support, and health: A research agenda,” by S. Lee, A. Arozullah, and Y. Cho, 2004, *Social Science & Medicine*, 58(7), p.1312.

## Methods

### Study Design

A cross-sectional study design was employed. Adult patients with breast cancer with positive human epidermal growth factor receptor 2 (HER2+) who received care (chemotherapy/HER2-directed therapy) at 12 oncology clinics in Texas and had scheduled office appointments between August and October 2018 were approached to participate in the study by clinic staff during clinic visits. Patients who were HER2+ were the target convenience sample because the clinics had a large population of such patients who were likely to have clinic visits for chemotherapy or follow-up. The participating oncology clinics were part of a larger practice with 210 locations in Texas and Oklahoma. This practice has a community-based approach to oncology care and adopts value-based care models such as the Oncology Care Model (OCM) that are designed to improve cancer patient care and the treatment experience. Specifically, these care models are designed to assist patients with better understanding their illness and treatment, managing treatment side effects, and avoiding hospital and emergency room visits when necessary (Texas Oncology, 2019a, 2019b).

### Measures

Patients who expressed willingness to participate completed a survey during their office visit that consisted of the 6-item cancer health literacy tool (CHLT-6), the 13-item patient activation measure (PAM-I 3), and the 27-item functional assessment of cancer therapy (FACT-G v.4). In addition, single items were used to measure the number of breast cancer-related emergency department (ED) visits and hospitalizations, as well as clinical and demographic patient characteristics (Kanu, 2019).

Prior research has found no evidence of an association between general health literacy (measured with the Test of Functional Health Literacy and the Rapid Estimate of Adult Literacy in Medicine) and engagement in health decisions for patients with cancer (Dumenci et al., 2014). Consequently, cancer health literacy (CHL) was measured using the CHLT-6 in this study because there is evidence that it is highly accurate in identifying patients with limited CHL who are less likely to engage in health decisions compared to those with adequate CHL (Dumenci et al., 2014). It identifies patients' CHL level based on responses to six items, including “If a patient has stage 1 cancer, it means the cancer is ___” and “Sally will get radiation therapy once a day, Monday through Friday. If Sally has therapy for 4 weeks, how many times will she get radiation therapy?” Each CHLT-6 item has only one correct response option. Correct responses are scored as one whereas incorrect responses are scored as zero. The 64 possible response patterns for the six items each have a specific probability of adequate and inadequate CHL that sum up to unity (Kanu, 2019). A patient's CHL is determined by selecting the cancer health literacy with the higher probability based on his or her response pattern to the six items. For example, a response pattern of 111011 to the six items has a 0.17 probability of limited CHL and a 0.83 probability of adequate CHL. Therefore, a patient with this response pattern will be considered to have adequate CHL (Dumenci et al., 2014).The PAM-13 was modified by replacing “health condition” with “breast cancer” where applicable and used to measure disease and self-care knowledge/ability in patients with breast cancer. Total PAM scores range from 0 to 100, with higher scores indicating greater patient activation. Each patient was grouped into one of the four levels of activation based on their total PAM score (Hibbard et al., 2005).

The 27-item FACT-G v.4, which was originally validated in a mixed cancer patient sample that included patients with breast, colorectal, and lung cancers, was used to measure HRQoL. In addition to test-retest reliability and responsiveness, it can discriminate between patients based on disease stage, performance status rating, and hospitalization status (Cella et al., 1993). The four FACT-G domains assess physical well-being, social/family well-being, emotional well-being, and functional well-being. Each item has response options on a 5-point Likert scale ranging from 0 (*not at all*) to 4 (*very much*), which are used to generate scores for each subscale. The four subscale scores are summed to obtain the total FACT-G v.4 score, which ranges from 0 to 108, with higher scores indicating greater HRQoL (Cella et al., 1993). Two open-ended items were used to capture resource use because it was not possible to gain access to this information from medical records: “Within the last 30 days, how many times have you had to visit the emergency department due to a breast cancer complication?” and “Within the last 30 days, how many times have you been hospitalized due to a breast cancer complication?” A recall period of 30 days was used to minimize recall bias.

Demographic and clinical characteristics of study participants including age, gender, ethnicity, educational level, stage of breast cancer at diagnosis, type(s) of breast cancer treatment(s) received, and comorbidities were measured with single items. Most patients received multiple types of breast cancer treatment and had multiple comorbidities; therefore, responses to these items were summed to obtain a composite score for the number of treatment types received and the number of comorbidities, respectively (Kanu, 2019).

### Data Analyses

All variables were analyzed descriptively by calculating means and standard deviations for continuous/interval variables as well as frequencies and percentages for categorical/nominal variables. Bivariate analyses were conducted to assess the relationships between cancer health literacy, patient activation, and demographic/clinical patient characteristics. Multiple regression was used to predict HRQoL with CHL and patient activation as the independent variables. In building a parsimonious regression model, all clinical/demographic variables that were not related to HRQoL in bivariate analysis were excluded from the model. Of the 10 clinical/demographic factors, the only two retained were education and number of treatment types received. Data analyses were conducted using SAS 9.4 software. This study was approved by the University of Texas Institutional Review Board.

## Results

### Participation Rate and Sample Characteristics

Almost 90% (*n* = 146) of 164 eligible patients who were offered participation in the study by clinic staff consented to participate and completed the survey. The mean age of participants was 57.1 ± 10.8 years, and all but one were female (99.3%). The majority of participants were White (71.7%), married or in a relationship (69.5%), had at least a college degree (52.8%), had private insurance (59.7%), and had an annual household income of more than $50,000 (66.2%). Clinically, there was an almost equal distribution of study participants from cancer stage 1 to stage 4, and about one-half were diagnosed within the last year (*n* = 73). Most patients (73.3%) had at least one comorbidity, with hypertension and hypercholesterolemia being the most common comorbidities (Kanu, 2019) (**Table 1**).

**Table 1:**
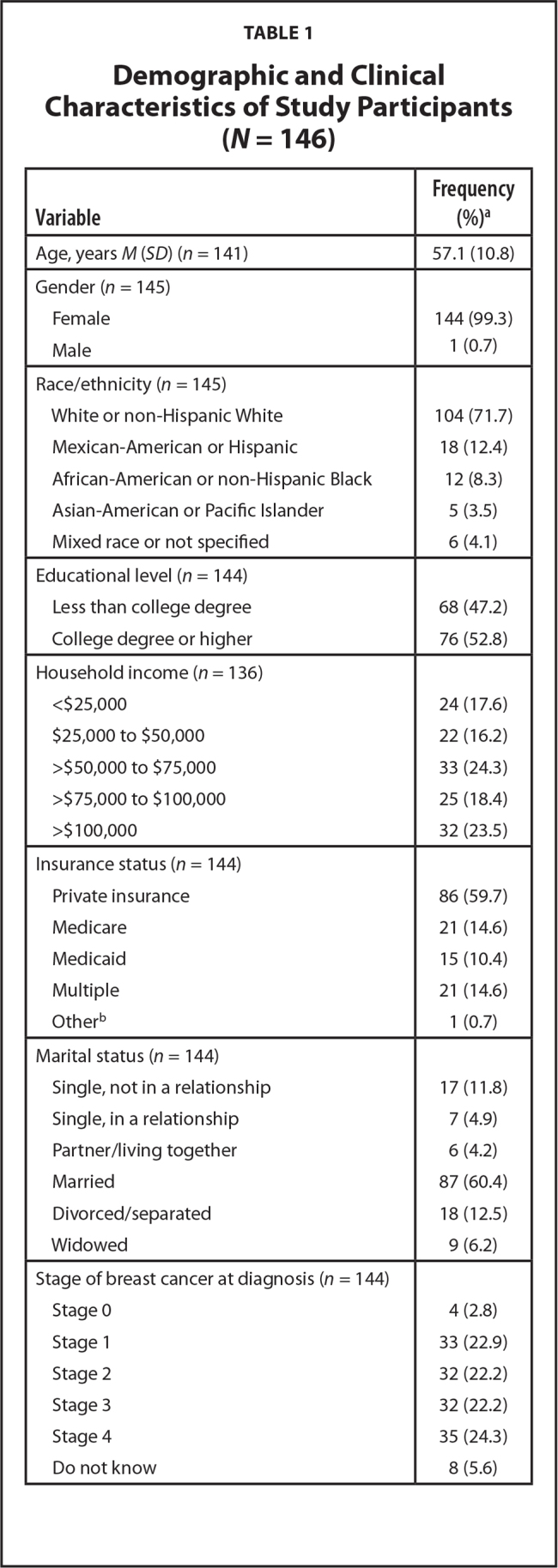

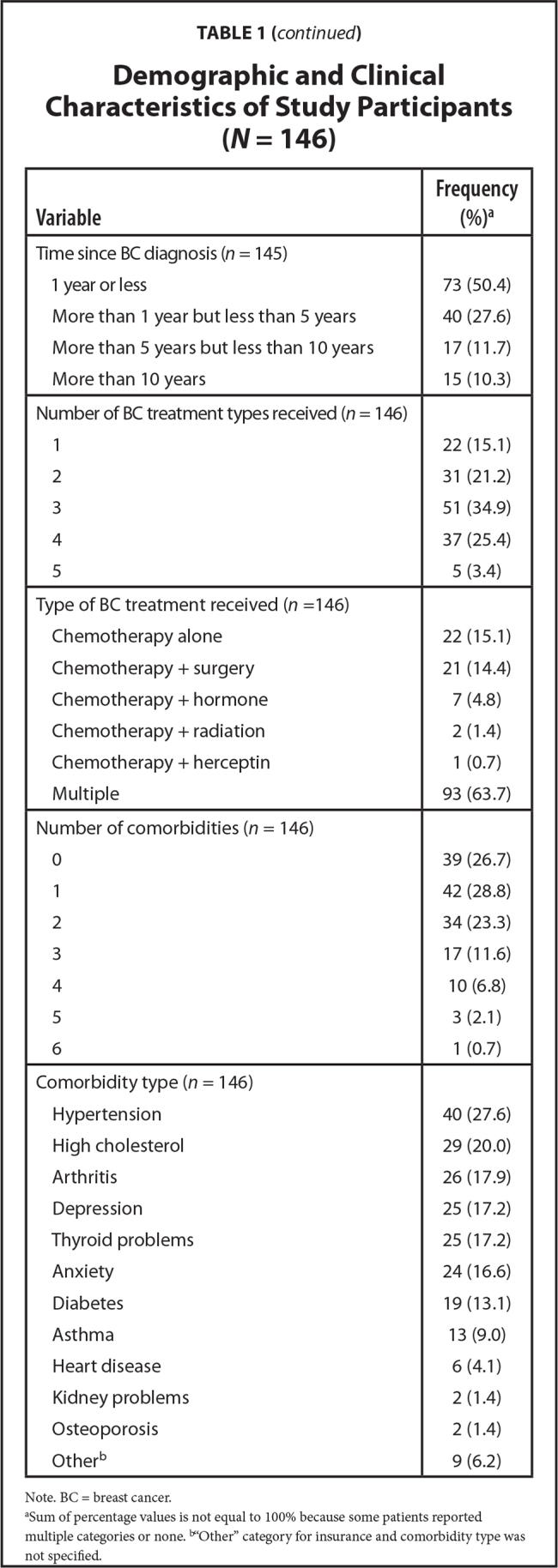
Demographic and Clinical Characteristics of Study Participants (*N* = 146)

**Variable**	**Frequency(%)^a^**
Age, years *M* (*SD*) (*n*= 141)	57.1 (10.8)

Gender (*n*= 145)	
Female	144 (99.3)
Male	1 (0.7)

Race/ethnicity (*n*= 145)	
White or non-Hispanic White	104 (71.7)
Mexican-American or Hispanic	18 (12.4)
African-American or non-Hispanic Black	12 (8.3)
Asian-American or Pacific Islander	5 (3.5)
Mixed race or not specified	6 (4.1)

Educational level (*n*= 144)	
Less than college degree	68 (47.2)
College degree or higher	76 (52.8)

Household income (*n* = 136)	
<$25,000	24 (17.6)
$25,000 to $50,000	22 (16.2)
>$50,000 to $75,000	33 (24.3)
>$75,000 to $100,000	25 (18.4)
>$100,000	32 (23.5)

Insurance status (*n*= 144)	
Private insurance	86 (59.7)
Medicare	21 (14.6)
Medicaid	15 (10.4)
Multiple	21 (14.6)
Other^b^	1 (0.7)

Marital status (*n*= 144)	
Single, not in a relationship	17 (11.8)
Single, in a relationship	7 (4.9)
Partner/living together	6 (4.2)
Married	87 (60.4)
Divorced/separated	18 (12.5)
Widowed	9 (6.2)

Stage of breast cancer at diagnosis (*n*= 144)	
Stage 0	4 (2.8)
Stage 1	33 (22.9)
Stage 2	32 (22.2)
Stage 3	32 (22.2)
Stage 4	35 (24.3)
Do not know	8 (5.6)

Time since BC diagnosis (*n*= 145)	
1 year or less	73 (50.4)
More than 1 year but less than 5 years	40 (27.6)
More than 5 years but less than 10 years	17 (11.7)
More than 10 years	15 (10.3)

Number of BC treatment types received (*n*= 146)	
1	22 (15.1)
2	31 (21.2)
3	51 (34.9)
4	37 (25.4)
5	5 (3.4)

Type of BC treatment received (*n*=146)	
Chemotherapy alone	22 (15.1)
Chemotherapy + surgery	21 (14.4)
Chemotherapy + hormone	7 (4.8)
Chemotherapy + radiation	2 (1.4)
Chemotherapy + herceptin	1 (0.7)
Multiple	93 (63.7)

Number of comorbidities (*n*= 146)	
0	39 (26.7)
1	42 (28.8)
2	34 (23.3)
3	17 (11.6)
4	10 (6.8)
5	3 (2.1)
6	1 (0.7)

Comorbidity type (*n*= 146)	
Hypertension	40 (27.6)
High cholesterol	29 (20.0)
Arthritis	26 (17.9)
Depression	25 (17.2)
Thyroid problems	25 (17.2)
Anxiety	24 (16.6)
Diabetes	19 (13.1)
Asthma	13 (9.0)
Heart disease	6 (4.1)
Kidney problems	2 (1.4)
Osteoporosis	2 (1.4)
Other^b^	9 (6.2)

Note. BC = breast cancer.

a Sum of percentage values is not equal to 100% because some patients reported multiple categories or none.

b “Other” category for insurance and comorbidity type was not specified.

### Health Literacy

The majority of study participants had a high probability (≥0.7 of 1.0) for having adequate CHL (92%; *n* = 134) having answered most of the CHLT-6 items correctly. There were significant relationships between CHL and household income (*p* = .02) and educational level (*p* = .01). Patients with higher incomes and more formal education showed adequate CHL compared to their counterparts.

### Patient Activation

Patient activation was relatively high (mean score: 65.9 ± 15.7; range: 34.2 to 100), and most patients with breast cancer (68%; *n* = 99) were in the higher activation levels (PAM level 3 or 4). Patient activation was significantly higher (*p* < .01) in people who were White (68.9 ± 16) compared to those who were Black (54.5 ± 6.9) or Hispanic (58.3 ± 10.7). The PAM had a high internal consistency as indicated by the Cronbach's coefficient alpha of 0.88. There was no significant difference (p = .62) in mean patient activation scores between patients with limited CHL (63.7 ± 10.4) and those with adequate CHL (66.1 ± 16.1) based on an independent groups *t*-test (Kanu, 2019).

### Health Outcomes

Study participants had a high average HRQoL score (82.6 ± 16.1) as measured by the FACT-G (possible range 0 to 108), with emotional well-being having the highest domain average (19.6 ± 3.4 of 24) and functional well-being having the lowest domain average (20.1 ± 5.7 of 28). The Cronbach's coefficient alpha of the overall FACT-G score in the study sample was 0.92 (0.7–0.9 for domains). There were very few reports of an ED visit (n = 5, 3.4%) or hospitalization (*n* = 2, 1.4%) in the last 30 days due to a breast cancer complication. Consequently, no bivariate/multivariate analyses were conducted on these health outcomes.

### HLS Framework

The overall model predicted a significant amount of variance in HRQoL (*F* = 10.31; *df* = 4,139; *p* < .0001), which supports the overall predictive validity of the HLS framework in explaining HRQoL (**Table 2**). Patient activation (*p* < .01), educational level (*p* = .04), and number of treatment types received (*p* = .02) were significant positive predictors of HRQoL and accounted for 23% of the variation in HRQoL (*R*^2^ = 0.23), with an adjusted *R*^2^ of 21% (*R*^2^ = 0.21). However, CHL (*p* = .77) was not a significant predictor (Kanu, 2019).

**Table 2:**
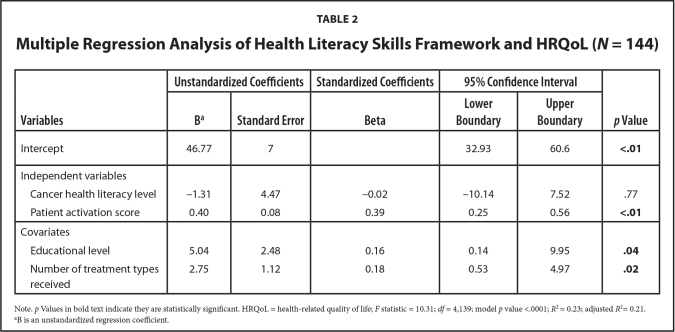
Multiple Regression Analysis of Health Literacy Skills Framework and HRQoL (*N* = 144)

**Variables**	**Unstandardized Coefficients**		**Standardized Coefficients**	**95% Confidence Interval**		***p* Value**

	**B^a^**	**Standard Error**	**Beta**	**Lower Boundary**	**Upper Boundary**	
Intercept	46.77	7		32.93	60.6	**<.01**
Independent variables						
Cancer health literacy level	−1.31	4.47	−0.02	10.14	7.52	.77
Patient activation score	0.40	0.08	0.39	0.25	0.56	**<.01**
Covariates						
Educational level	5.04	2.48	0.16	0.14	9.95	**.04**
Number of treatment types received	2.75	1.12	0.18	0.53	4.97	**.02**

Note. *p* Values in bold text indicate they are statistically significant. HRQoL = health-related quality of life; *F* statistic = 10.31; *df* = 4,139; model *p* value <.0001; *R* = 0.23; adjusted *R* = 0.21.

a B is an unstandardized regression coefficient.

## Discussion

Breast cancer can now be considered as a chronic condition due to improvements in detection, diagnosis, and treatment (McCorkle et al., 2011). Patients with chronic diseases, including breast cancer, are being increasingly expected to play an active role in managing their care. Study participants were found to have high levels of CHL and patient activation, which was not surprising as most of them were socioeconomically advantaged. Socioeconomic status is known to be positively associated with these constructs (Hibbard et al., 2004; Kutner et al., 2006). Unfortunately, only a few patients reported cancer-related ED visits and hospitalizations; therefore, the relationship of resource use with health literacy and patient activation could not be assessed.

The HLS framework was useful in explaining HRQoL in patients with breast cancer, with patient activation, educational level, and number of treatment types received explaining almost one-quarter of the variance in patients' HRQoL. However, CHL was not shown to be related to HRQoL, neither indirectly through patient activation as posited in the HLS framework, nor directly. This could have resulted from the dichotomous nature of the CHL instrument used and/or the homogeneity of responses, as most patients had adequate CHL. Therefore, there was insufficient evidence to support or refute the potentially important relationship between health literacy and patient activation and HRQoL in patients with breast cancer.

The relationship between patient activation and HRQoL found in this study is supported by similar findings in other studies. Magnezi et al. (2014) reported a significant positive correlation between patient activation and the total HRQoL scores as measured by Short Form-12 Health Survey (SF-12), as well as the physical and mental health subscale scores in 278 adults age 18 to 85 years at primary care clinics. Participants generally had a high HRQoL (32.1 ± 8.3, range of 12 to 44) (Magnezi et al., 2014). A study by Blakemore et al. (2016), showed that patient activation was significantly lower in people with poor health literacy (as measured by the Single Item Literacy Screener) and higher in those with good HRQoL (as measured by the 5-level EuroQol 5D health utility index) in a large cohort of adults age 65 years and older.

The current study also added support to the significance of number of treatment types and educational level in the HRQoL of patients with breast cancer. Similar findings from other studies show that sociodemographic and clinical characteristics are associated with HRQoL in oncology practice. For example, a study by Daldoul et al. (2018) examined the relationship of quality of life and sociodemographic, clinical, and treatment factors in 70 patients with a confirmed diagnosis of breast cancer. Results showed that HRQoL (measured by the Short Form-36 health survey) was significantly associated with receipt of chemotherapy as well as its side effects (*p* = .01). However, age, marital status, stage of cancer, comorbidities, and other treatment types (surgery, radiotherapy, or hormone therapy) were not significantly associated with HRQoL (Daldoul et al., 2018). Another study showed that patient characteristics, including educational level and employment status, were significant predictors for HRQoL in a sample of 608 patients with breast cancer (Chen et al., 2018).

To our knowledge, this is the first study to explore these relationships in patients with breast cancer. In addition to improved HRQoL, higher patient activation levels have been associated with improved health outcomes and lower resource use in other chronic conditions, including diabetes, heart failure, and asthma (Begum et al., 2011; Mosen et al., 2007). This study supports positive associations in patients with breast cancer as well. Highly activated cancer patients tend to be better informed and more proactive about managing their condition (Hibbard et al., 2007). They are also more likely to understand their diagnosis, efficiently manage side effects, feel sufficiently informed, and have their values reflected in their treatment plans. Furthermore, poorly activated patients with cancer tend to be less satisfied with their care (Hibbard et al., 2017). Therefore, cancer care providers should consider assessing patient activation at the beginning of cancer care and subsequently encouraging their patients with breast cancer to participate in managing their care as a quality metric of care by providing relevant information to patients based on their activation level. In instances where there is a time constraint for the clinician to address patient concerns, providing general reference material for typical concerns of patients with breast cancer with recommended coping techniques can be an alternative to improving patient involvement in their care. In addition, more attention could be given to patients with less formal education who might find it particularly difficult to understand clinical terms by minimizing medical/technical terms in written and spoken communication.

## Study Limitations

Some limitations should be considered when interpreting study findings. Only self-reported data were used, which could have been subject to recall bias. Also, given the descriptive and cross-sectional nature of this study, causality cannot be inferred. In addition, selection bias could have occurred as a result of convenience sampling. Furthermore, the study sample was from multiple clinics within a Texas-based oncology group that implements value-based care models. Consequently, the study results may only be generalizable to patients with breast cancer who receive care from similar practices. Finally, the patient activation score of study participants pertained to their knowledge/ability to self-manage their breast cancer care and may not necessarily indicate how they would manage other comorbidities/chronic conditions.

## Suggestions for Future Research

Future research should consider using the longer 30-item CHL tool or a similar instrument that measures health literacy along a continuum and can assess degrees of limitedness or adequacy of health literacy.

## Conclusion

This study lends support to the use of the HLS framework in predicting HRQoL in patients with HER2+ breast cancer. Findings show that patient activation, which is a modifiable factor, was a significant and positive predictor of quality of life. Cancer health literacy was not significantly associated with patient activation nor predictive of HRQoL, although the homogeneity of CHL levels among study participants could have affected the results. Further assessments of health literacy and patient activation with quality of life as well as other health outcomes in more diverse populations of patients with breast cancer are warranted.
